# Machine learning methods to predict outcomes of pharmacological treatment in psychosis

**DOI:** 10.1038/s41398-023-02371-z

**Published:** 2023-03-02

**Authors:** Lorenzo Del Fabro, Elena Bondi, Francesca Serio, Eleonora Maggioni, Armando D’Agostino, Paolo Brambilla

**Affiliations:** 1 Department of Pathophysiology and Transplantation, University of Milan, Milan, Italy; 2 Department of Neurosciences and Mental Health, Fondazione IRCCS Ca’ Granda Ospedale Maggiore Policlinico, Milan, Italy; 3 Department of Health Sciences, University of Milan, Milan, Italy; 4 Department of Mental Health and Addiction, ASST Santi Paolo e Carlo, Milan, Italy; 5 Department of Electronics, Information and Bioengineering, Politecnico di Milano, Milan, Italy

**Keywords:** Schizophrenia, Predictive markers

## Abstract

In recent years, machine learning (ML) has been a promising approach in the research of treatment outcome prediction in psychosis. In this study, we reviewed ML studies using different neuroimaging, neurophysiological, genetic, and clinical features to predict antipsychotic treatment outcomes in patients at different stages of schizophrenia. Literature available on PubMed until March 2022 was reviewed. Overall, 28 studies were included, among them 23 using a single-modality approach and 5 combining data from multiple modalities. The majority of included studies considered structural and functional neuroimaging biomarkers as predictive features used in ML models. Specifically, functional magnetic resonance imaging (fMRI) features contributed to antipsychotic treatment response prediction of psychosis with good accuracies. Additionally, several studies found that ML models based on clinical features might present adequate predictive ability. Importantly, by examining the additive effects of combining features, the predictive value might be improved by applying multimodal ML approaches. However, most of the included studies presented several limitations, such as small sample sizes and a lack of replication tests. Moreover, considerable clinical and analytical heterogeneity among included studies posed a challenge in synthesizing findings and generating robust overall conclusions. Despite the complexity and heterogeneity of methodology, prognostic features, clinical presentation, and treatment approaches, studies included in this review suggest that ML tools may have the potential to predict treatment outcomes of psychosis accurately. Future studies need to focus on refining feature characterization, validating prediction models, and evaluate their translation in real-world clinical practice.

## Introduction

Schizophrenia (SCZ) is a major chronic psychiatric disorder that represents one of the top eight causes of disability worldwide [1], leading to demanding social, professional, and economic consequences [2, 3]. SCZ typically develops during early adulthood. It is characterized by positive, negative, and cognitive symptoms. While positive symptoms are characterized by hallucinations, delusions, and formal thought disorder, negative symptoms consist of a lack of volition and emotiveness with reduced speech output. Cognitive symptoms are characterized by cognitive deterioration in all domains of neuropsychological function [3].

Although the pathogenesis of SCZ is still unknown, multiple strands of evidence indicate that it is a progressive neurodevelopmental disorder [4]. Recent advances in research have suggested that SCZ is a multifactorial disorder with a combination of genetic and environmental risk factors involved in its pathogenesis. In recent years, genome-wide association studies have identified hundreds of genetic loci that were associated with SCZ proving its polygenic disorder nature [5]. Moreover, neuroimaging studies have played a central role in providing abundant evidence of structural and functional brain abnormalities in patients with SCZ at different phases of the disorder [6–9].

SCZ requires long-term treatment that is commonly based on antipsychotic medications, which are primarily indicated for the treatment of SCZ and psychotic disorders [10]. First-generation antipsychotics (FGAs), also known as typical antipsychotics, were developed in the 1950s [11]. The efficacy of this pharmacological class depends on its ability to reduce dopamine function by blocking the dopamine D_2_ family of postsynaptic receptors [3]. However, the occurrence of adverse effects associated with FGAs, in particular debilitating extrapyramidal side effects, led to the introduction of second-generation antipsychotics (SGAs), also known as atypical antipsychotics. The SGAs are potent 5-HT_2a_ receptor antagonists and relatively weaker dopamine D_2_ antagonists and are associated with a substantially lower risk of neurologic adverse effects [12].

Treatment choices for patients with SCZ and psychotic disorders are currently based on treatment guidelines broadly depending on clinical conditions and symptom classification without reference to the patients’ biological background [13]. In this context, the identification of predictors of treatment response in patients with SCZ is a task of substantial importance to help clinicians make informed treatment initiation and personalize treatment decisions [14]. Specifically, over the last decades researchers have tried to identify specific factors involved in treatment response, leading to studies focusing on multiple variables, such as clinical, neuroimaging, and genetic characteristics, to create prognostic prediction models [15].

In recent years, machine learning (ML) approaches have been suggested to be a promising innovative tool with the potential to develop accurate and generalizable treatment response predictions about individuals with psychiatric disorders [15, 16]. ML is a subfield of artificial intelligence broadly defined as a computational strategy which employs algorithms that automatically determine methods and parameters learning from complex data to reach an optimal prediction [16–18]. Before ML analysis, a rigorous collection of relevant data and pre-processing steps are performed, then prediction models are trained and tested during the learning process [18]. Recently, ML research has used the power of large-scale, multidimensional databases and advanced biological data sources to develop prediction models for diagnostic, prognostic, and treatment selection procedures [15, 16]. Interestingly, ML techniques have been also used to predict treatment outcomes in patients with psychiatric disorders such as depression, showing good accuracies [19–21].

For SCZ, important advances have been made toward the identification of clinical and biological predictors of treatment response, especially in studies using large multisite treatment databases containing prospective data of individuals with an early course of psychosis [22, 23]. Indeed, ML techniques could help direct the early implementation of targeted interventions that have been shown to result in better clinical and functional outcomes for more vulnerable individuals [24]. Thus, mainly clinical [23] and neuroimaging data [25, 26] have been used as potential predictors of treatment outcomes in SCZ.

Given that SCZ is associated with magnetic resonance imaging (MRI) signal abnormalities, and that these have been associated with symptom profiles as well as outcome, neuroimaging measures have been considered promising biological markers for treatment outcome [26]. Especially in MRI studies, important findings have been discovered. Indeed, findings from structural MRI (sMRI) studies indicate that response to antipsychotics is associated with altered brain volumes in specific brain regions, while functional MRI (fMRI) studies observed increased baseline brain activity and connectivity in treatment responders [25].

Despite the increasing number of studies published in this area over recent years, the impact of ML on treatment response prediction in patients with psychosis is still unclear. No study to our knowledge has comprehensively reviewed the advancements and challenges of ML approaches in the development of therapeutical predictors in SCZ to date. In this context, this study aims to provide a comprehensive literature review of current knowledge about ML methodologies applied for the prediction of antipsychotic treatment response in individuals with early and chronic course of SCZ.

## Methods

For the purpose of this review, a literature search was performed on the Pubmed database following the PRISMA guideline recommendations [27]. The following search words were used: “schizophrenia” OR “psychosis”, “treatment” OR “antipsychotics”, “machine learning” OR “prediction”. The electronic search was completed on all available years until March 15, 2022. Records were screened after the removal of duplicates based on the title, abstract, and full text. The selection process was conducted by two independent researchers. Disagreements were resolved by a third independent investigator.

The inclusion criteria for the studies were the following: (1) English language, (2) clinical trials, (3) human studies, (4) inclusion of subjects clinically diagnosed with SCZ or non-affective psychotic disorders, (5) use of treatment with antipsychotics, and (6) use of ML techniques to predict clinical outcome. For each publication, the following variables were extracted: sample characteristics, clinical information, ML analyses performed, performance measures, and main results.

## Results

**Fig. 1:**
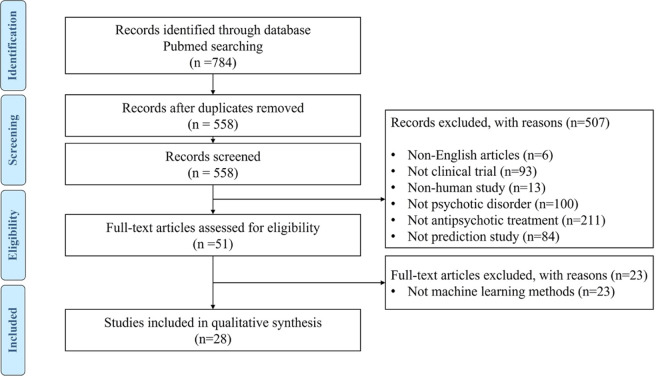
Flow chart of study selection.

The database literature search resulted in 784 articles, which were screened for eligibility. Among them, 226 studies were duplicates. Then, 507 articles were excluded based on title and abstract review. The full text of the remaining 51 papers was checked for eligibility and 23 of them were excluded. Overall, a total of 28 articles were included in this review (Fig. 1). The main characteristics and findings of the included studies are summarized in Table 1.

**Table 1:** Summary of the included original studies.

Study	Sample (M/F) Age (years) mean ± SD	Type of psychosis	Psychotic symptom severity (baseline)	Treatment	Other medications	Features and Outcomes	Analyses	Results
Ambrosen et al., 2020 [28] (1)	138 SCZ (94/44), 25.36 ± 5.88	FEP	PANSS: 80.32 ± 16.45	Atypical AP. Dosage was increased until a clinical antipsychotic effect was evident.	No antypsychotics or methylphenidate, antidepressant treatment within 1 month prior to baseline examinations.	*Features:* sMRI EEG Cognitive tests *Covariates*: age, sex, cohort, and handedness. *Outcomes:* Short-term response: change in PANSS Long-term response: binary classification based on clinical data	**Single algorithm approach.** - *short-term response*: linear regression algorithms, SVMs, with different kernels, Gaussian Processes, regression trees, generalized linear models, ensemble regression algorithms, and RF; - *long-term response*: logistic regression, Naïve Bayes, RF, decision trees, ensemble of trees, SVMs with different kernels, and k-nearest neighbor. **Ensamble approach**, implemented using auto-sklearn, testing two parameters on simulated data: time limit (T) (20, 60, 180s) and maximum number of algorithms included (N) (1, 4, 40).	**Single algorithm approach:** - *short-term response:* BACC = 50.30% - *long-term response*: not significant **Ensamble approach** *- long-term response:* BACC = 50.0% *- short-term response*: not significant *-Meaningful predictors*: cognitive data.
Anderson et al., 2017 [40]	308 PP-C (219/89), 42.0 ± 12.7 174 PP-N (125/49), 39.6 ± 12.2 281 OAT (181/100), 42.1 ± 13.4	Chronic SCZ	PANSS	PP-C initiated PP more than 8 weeks prior enrollment; PP-N initiated PP within 8 weeks of enrollment; OAT initiated an atypical oral AP medication or combination within 8 weeks or prior to enrollment.	Not reported	*Features:* Demographic information; Clinical characteristics; Behavioral/lifestyle factors; Healthcare resource utilization; Patient-reported outcomes *Outcomes: clinical remission*	ML platform to identify meaningful predictors for treatment outcomes; Multivariable Cox or generalized linear regression models to evaluate effects of treatment status on study outcomes, using as covariates meaningful predictors.	*-Remission*, AUC = 0.75. *-Meaningful predictors*: PP use and the absence of psychiatric comorbidities
Blessing et al., 2019 [29] (1)	27 FEP (12/15), 24.11 ± 7.19	FEP	BPRS: 52.56 ± 13.2	Chlorpromazine for 8 weeks: 488 ± 275.88 (mg/d)	Psychotropic naive at baseline.	*Features:* rs-fMRI *Outcomes:* Responders vs nonresponders (based on the BPRS total score)	RF	-*Treatment response:* AUC = 0.95; ACC = 0.89 -*Meaningful predictors:* FC between anteromedial hippocampal and right SFG, left PreCG, right posterior InsOperc, and left PCG
Cao et al., 2018 [30] (1)	43 FEP (19/24), 28.3 ± 9.9	FEP	PANSS	Risperidone for 10 weeks. Dose was increased to 3–6 mg/d for 1 week, than kept at the same level.	Chloral hydrate or lorazepam for insomnia, and benzhexol hydrochloride as antiparkinsonian agents for extrapyramidal symptoms, as needed. No other concomitant psychotropic medications were used during the study.	*Features:* rs-fMRI *Outcomes:* Percentage drop of PANSS total scores	SVR	*-Treatment response:* ACC = 0.83 -*Meaningful predictors:* FC of superior temporal cortex
Ciprian et al., 2020 [41]	57 SCZ (37/20), 36.95 ± 9.22	Chronic SCZ	PANSS	Clozapine for at least 1 year. Mean dose: 347 mg.	Not reported	*Features:* EEG *Outcomes:* Most responder vs less responder (based on change of PANSS)	SVM, RF, LDA	*-Treatment response:* ACC = 0.96
Cui, LB. et al., 2021a [48]	*Dataset 1:* 47 responders (26/21), 25.1 ± 5.7 38 non-responders (22/16), 26.0 ± 7.0 *Dataset 2:* 41 responders (27/14), 21.9 ± 5.5, 22 non-responders (11/11), 26.0 ± 9.1, *Dataset 3 (independent validation set)* 28 responders 28 non-responders	FEP Chronic SCZ	PANSS *Dataset 1* responders: 97.6 ± 20.0 non-responders: 91.1 ± 14.3 *Dataset 2* responders: 83.0 ± 21.8 non-responders: 87.9 ± 12.2	**(FGA/SGA), dose [mg/d]** *Dataset 1*: responders (12/47), 11.4 ± 4.7 non-responders (9/38), 10.9 ± 4.9 *Dataset 2:* responders (7/41), 12.0 ± 5.5 non-responders (4/21), 11.5 ± 6.5. *Dataset 3:* 15.65 ± 6.57	Not reported	*Features:* sMRI fMRI *Outcomes:* Responder vs non-responder (based on change of PANSS)	10-fold SVM	-*Treatment response:* ACC = 0.85 using only Dataset 1 and Dataset 2 ACC = 0.69 using validation set (Dataset 3) -*Meaningful predictors:* higher accuracy was obtainined combining sMRI and fMRI features.
Cui, LB. et al., 2021b [49] (1)	61 responders (40/21), 24 ± 6 48 non-responders (26/22), 27 ± 8	FEP Chronic SCZ	PANSS responders: 90 ± 20 non-responders: 89 ± 14	Majority received SGA, minority FGA Dose [mg/d] *responders*, 10 ± 4 *non-responders*, 10 ± 4	Not reported	*Features:* sMRI *Outcomes:* Responder vs non-responder (based on the change in PANSS)	RF	*-Treatment response:* ACC = 0.75 *-Meaningful predictors*: thalamus-based radiomic features
Cui, X. et al., 2021 [31]	44 FEP (28/16), 23.45 ± 4.24	FEP	PANSS: 90.70 ± 11.17	Olanzepine for 8 weeks. Dose: 18.30 ± 5.17 (mg/d)	Drug-naive	*Features:* sMRI *Outcomes:* Reduction ratio (RR) of PANSS total scores	SVR	-*Treatment response*: significant prediction -*Meaningful predictors*: GMV of superior temporal gyrus
Ebdrup et al., 2019 [32] (1)	46 FEP (28/18), 25.0 ± 5.6	FEP	PANSS: 83.5 ± 17.2	6 weeks of amisulpride. Dose: 248.4 ± 140.6 (mg/d)	Antipsychotic naive	*Features:* EEG sMRI DTI Neurocognitive tets (each set of feature were studied separately) *Outcomes:* Remission vs non-remission (using the Andreasen criteria)	6 different ML approaches	*-Remission:* none of the modalities predicted symptom remission.
Fonseca de Freitas et al., 2022 [42]	242 SCZ (162/80), 35.9 (28.2–44.0)	Chronic SCZ	Not reported.	3 months of clozapine	165 patients (68.2%) had received a depot medication (LAI) in the 6 months before clozapine initiation	*Features:* Sociodemographical Clinical potential predictors of response to clozapine *Outcomes:* CGI scale	LASSO	*-Treatment response*: model’s optimism-corrected calibration slope = 1.37 *-Meaningful predictors*: severity of illness at baseline, female gender, and comorbid mood disorder
Homan et al., 2019 [50]	82 SCZ (59/23), 21.6 ± 5.5	FEP early-diagnosed SCZ	BPRS: 42.7 ± 7.5	Aripiprazole (5–30 mg/d) vs risperidone (1–6 mg/d) for 12 weeks.	Medication naive or medicated	*Features:* sMRI *Outcomes:* BPRS at different time points	PLS	*-Treatment response:* significant prediction -*Meaningful predictors:* orbito- and prefrontal, superior temporal, precentral, and middle cingulate cortex.
Khodayari-Rostamabad et al., 2000 [47]	*Training set* 23 SCZ (12/11), 41.2 ± 8.4 *Test set* 14 SCZ (8/6), 35.7 ± 10	chronic SCZ	PANSS only for training set after treatment	Clozapine. - training set: 344.6 ± 157 (mg/d) - test set: 396.4 ± 101 (mg/d)	Not reported	*Features:* EEG *Outcomes:* Responder vs non-responder (based on change in QCA-score)	KPLSR	*-Treatment response:* ACC = 0.85
Koutsouleris et al., 2016 [23]	*Training set* 334 FEP (188/146), 26.1 ± 5.6 *Test set* 108 FEP (72/36), 25.6 ± 5.1	FEP	PANSS: training set: 88.8 ± 20.6 test set: 88.2 ± 19.6	Haloperidol, Olanzapine, Amisulpride, Quetiapine, Ziprasidone.	Not reported	*Features:* Sociodemographical features *Outcomes:* Good vs bad outcome (based on Global Assessment of Functioning scores)	Different classifiers (linear and nonlinear SVM, decision trees, univariate and multivariate logistic regression)	-*Treatment response*: BACC = 0.72 *-Meaningful predictors*: unemployment, poor education, functional deficits, and unmet psychosocial needs
Legge et al., 2020 [38] (2)	561 TRP (361/200) 509 non-TRP (300/209)	Chronic SCZ Related psychotic disorders	Not reported.	TRP: patients who had responded to neuroleptics or had receveid clozapine treatment non-TRP: patients who had responded to any type of antypsichotic and had not received clozapine	Not reported.	*Features:* Sociodemographical features *Outcomes:* TRP measure (TRP if they had either been rated negatively for OPCRIT item 89 or had recieved clozapine)	Multivariate logisitc regression Conditional inference forest model	*-Treatment response*: ACC = 0.59 *-Meaningful predictors*: age of onset, poor premorbid social adjustment, family history of schizophrenia, lower premorbid IQ and poor premorbid work adjustment.
Li et al., 2019a [33]	*Training set* 32 FEP (16/16), 30.94 ± 8.25 *Test set* 44 FEP (28/16), 23.45 ± 4.24	FEP	PANSS training set: 77.38 ± 5.17 test set: 90–70 ± 11.17	Olanzapine for 8 weeks. - training set: 18.59 ± 8.25 - test set: 18.30 ± 5.17	Drug naive.	*Features:* rs-fMRI *Outcomes:* Reduction rate of PANSS	SVR	-*Treatment response:* significant prediction -*Meaningful predictors*: left ventromedial putamen functional activity
Li et al., 2019b [34]	32 FEP (16/16), 30.94 ± 8.25	FEP	PANSS: 77.38 ± 5.17	Olanzapine for 8 weeks, 18.59 ± 4.96 (mg/d)	Drug naive.	*Features:* rs-fMRI *Outcomes:* Reduction rate of PANSS	SVR	-*Treatment response:* significant prediction -*Meaningful predictors*: FC of bilateral anterior cingulate cortex
Li et al., 2021 [51] (3)	PSP positive group: 159 SCZ (101/58), 41.6 ± 17.3 PSP negative group: 391 SCZ (361/35), 50.96 ± 14.5	FEP Chronic SCZ	PANSS PSP positive group: 71.0 ± 18.5 PSP negative group: 59.1 ± 17.8	Atypical antypsichotics	Mood stablizers, used as features.	*Features:* Socio-demographical Clinical information *Outcomes:* Positive vs negative group (positive: ≥10 points in the PSP)	Different classifiers (logistic regression, stochastic gradient descendent, gradient boosting decision tree, extreme gradient boosting, and RF)	-*Treatment response:* AUC = 0.86 -*Meaningful predictors:* PSP score at baseline, mood stabilizers, PANSS total score.
Masychev et al., 2020 [43]	30 most responders SCZ (15/15), 35.81 ± 6.33 [median ± MAD] 30 least responders SCZ (22/10), 36.82 ± 5.34 [median ± MAD]	Treatment-resistant SCZ	PANSS most responders: 62 ± 7.5 [median ± MAD] least responders: 39 ± 9 [median ± MAD]	Clozapine for at least 1 year. Dose: - most responders: 350 ± 137.5 (mg/d) [median ± MAD] - least responders: 400 ± 100 (mg/d) [median ± MAD]	Not reported.	*Features:* EEG *Outcomes:* Most responders vs least responders (based on changes in PANSS)	Different classifiers (SVM, LDA, RF, and K-nearest neighbors)	*-Treatment response:* ACC = 0.89%
Podichetty et al., 2021 [44]	639 SCZ (473/166)	SCZ	PANSS: 36 ± 9	Atypical antypsichotics	Not reported.	*Features:* Socio-demographical *Outcomes:* Improvement vs non-improvement (based on changes of PANSS)	Different classifiers (SVM, logistic regression, RF, and naive Bayes)	*-Treatment response:* AUC = 0.7 -*Meaningful predictors:* PANSS subscales scores
Sarpal et al., 2016 [52] (1)	*Training set* 41 FEP *-* 24 responders (16/8), 21.2 ± 3.8 − 17 non-responders (13/4), 21.9 ± 5.9 *Independent set* 40 hospitalized chronic SCZ − 20 responders (15/5), 28.5 ± 11.0 *-* 20 non-responders (13/7), 28.0 ± 9.7	FEP Chronic SCZ	BPRS *Training set* responders: 44.1 ± 8.3 non-responders: 43.0 ± 8.2 *Independent set* responders: 19.9 ± 5.5 non-responders16.9 ± 5.7	FEP: atypical antipsychotics; hospitalized SCZ: typical or atypical antipshycotics.	FEP: simultaneous treatment with mood stabilizers or antidepressants was not allowed, though patients were treated with diphenhydramine or benztropine as needed for extrapyramidal symptoms, and lorazepam for akathisia, agitation, and anxiety. hospitalized SCZ: reported medications.	*Features:* rs-fMRI *Outcomes:* Responders vs non-responders (based on changes in CGI scale)	Cox regression	-*Treatment response:* significant prediction -*Meaningful predictors*: FC of the striatum
Shan et al., 2021 [53] (1)	20 SCZ (15/5), 22.75 ± 4.38	FEP Relapse SCZ	PANSS: 52.80 ± 5.11	Olanzapine for 8 weeks, 20.50 ± 1.54 (mg/d)	Drug naive.	*Features:* rs-fMRI *Outcomes:* Reduction rate of PANSS scores	SVR	-*Treatment response:* significant prediction -*Meaningful predictors:* FC of superior/middle medial prefrontal
Smucny et al., 2021 [39] (1) (4)	65 SCZ (49/16), 20.8 ± 3.3 18 BD (9/8), 21.6 ± 2.8	SCZ BD	BPRS: 42.7 ± 9.7	Atypical antipsychotics.	Not reported.	*Fetures:* fMRI (ROI and voxelwise) *Outcomes:* - Improvers vs not-improvers (based on a decrease higher than 20% of BPRS) - Continuous change of BPRS	Different classifiers (logistic regression, nB, SVM, K-nearest neighbor, AdaBoost, J48 decision tree, RF, DL)	-*Treatment response:* ACC = 0.70 using DL -*Meaningful predictors*: functional activation of dorsolateral prefrontal cortex
Soldatos et al., 2022 [35]	*Dataset 1* − 102 remission (64/58), 25.9 ± 7.5 − 77 non-remission (56/21), 25.7 ± 7.7 *Dataset 2* − 31 remission (16/15), 22.8 ± 3.7 − 70 non-remission (37/33), 24.3 ± 5.9	FEP	PANSS. *Dataset 1* remission: 93.9 ± 21.2 non-remission: 106.6 ± 25.2 *Dataset 2* remission: 70 ± 15.9 non-remission: 78.8 ± 14	Atypical antipsychotics.	Antipsychotic naive.	*Features:* Socio-demographical data *Outcomes:* Remission vs non-remission (based on the Andreasen PANSS remission criteria)	SVM	**Dataset 1** *Remission:* AUC = 71.45 **Both datasets (training and test)** *-Remission:* AUC = 67.74 -*Meaningful predictors*: Duration of Untreated Psychosis, Personal and Social Performance Scale, Global Assessment of Functioning, and PANSS scores.
Uematsu & Hisanobu, 1998 [45] (1)	40 SCZ − 24 poor response (24/0), 33.3 ± 7.3 − 16 good response (16/0), 30.4 ± 5.4	Chronic SCZ	BPRS poor response: 27.7 ± 10.6 good response: 17.6 ± 9.8	Neuroleptic drugs. Dose (chlorpromazine equivalent): - poor response: 621 ± 479 (mg/d) - good reponse: 237 ± 234 (mg/d)	Not reported.	*Features:* sMRI Socio-demographical data *Outcomes:* Drug response	Multiple regression	-*Treatment response:* significant prediction -*Meaningful predictors:* cerebellar vermis sizes, symptom severity rates, and duration of hospitalization
Veronese et al., 2021 [54]	*Dataset 1 (FEP)* − 13 responders (10/3), 24,4 ± 3.02 − 13 non-responders (12/1), 26.2 ± 5.78 *Dataset 2 (Non-responsive SCZ)* − 12 responders (6/6), 44 ± 11.9 − 12 non-responders (5/7), 45.7 ± 9.8	FEP Non-responsive SCZ	PANSS *Dataset 1:* responders: 74.23 ± 16.96 non-responders: 73.08 ± 14.63 *Dataset 2:* not reported	Atypical antipsychotics.	Dataset 1: unmedicated/minimally treated Dataset 2: only medicated patients	*Features:* PET *Outcomes:* Responders vs non-responders	Different classifiers (Bernoulli, SVM, RF, Gaussian processes)	*-Treatment response:* AUC = 0.89 -*Meaningful predictors*: brain function in striatal regions
Wang et al., 2022 [46]	20 responders (7/13), 25.22 ± 5.4 37 non-responders (20/17), 28.35 ± 7.3	SCZ	PANSS responders: 76.90 ± 8.3 non-responders: 79.21 ± 7.8	Atypical antipsychotics for 6 weeks) Chlorpromazine equivalent dosage (mg/d): - responders: 418.42 ± 280.6 - non-responders: 531.03 ± 367.9	Not reported.	*Features:* sMRI fMRI Plygenic risk score (PRS) *Outcomes:* Responders vs non-responders (based on changes of PANSS)	Gradient boosting	-*Treatment response:* ACC = 0.86 -*Meaningful predictors*: gray matter volume, ALFF, surface curvature
Wood et al., 2006 [36]	46 FEP (29/15), 21.6 ± 3.2	FEP	PANSS	Atypical antipsychotic.	Not reported.	*Features:* Proton magnetic resonance spectroscopy *Outcomes:* Clinical measures	Omnibus multivariate regression	-*Treatment response:* significant prediction -*Meaningful predictors:* *N*-acetylaspartate and choline-containing compounds to creatine and phosphocreatine ratio in prefrontal cortex
Wu et al., 2020 [37]	32277 FEP (48.8% males), 36.7 ± 14.3	FEP	Not reported.	Atypical antipsychotic.	Reported.	*Features:* Socio-demographic Clinical *Outcomes:* Treatment success vs treatment failure (treatment success: absence for 12 months of inpatient mortality and either hospitalization or change in treatment)	Super Learner	-*Treatment response:* -*Meaningful predictors:* age, psychotic symptom severity and the use of mood stabilizers or benzodiazepines,

Study also included HC for other anlayses.

Analyses were carried out on a subsample of 337 individuals with no missing data.

Study also included a comparison between comorbid and non-comorbid mood stabilizer groups.

Study also included a deep-learning classification model.

*HC* healthy controls, *SCZ* patients with schizophrenia, *FEP* patients with first-episode psychosis, *TRP* treatment-resistant psychosis, *PP* paliperidone palmitate, *PP-C* continuing users of PP *PP-N* new users of PP, *OAT* atypical oral antipsychotic therapy, *BD* bipolar disorder, *LAI* long-acting injectable, *AP* antipsychotic, *MAD* median absolute deviation,

*MRI* magnetic resonance imaging, *sMRI* structural MRI, *EEG* electroencephalography, *fMRI* functional MRI, *rs* resting-state, *DTI* diffusion tensor imaging, *fALFF* fractional amplitude of low-frequency fluctuation,

*RF* random forest, *AUC* area under the curve, *ACC* accuracy, *FC* functional connectivity, *MI* mutual information, *SVM* support vector machine, *LDA* linear discriminant analysis, *SVR* support vector regression, *LASSO* least absolute shrinkage and selection operator, *LR* linear regression, *nB* naive bayes, *PLS* partial least square, *KPLSR* kernelized partial least squares regression, *DL* deep learning,

*BACC* balanced accuracy, *NMSE* normalized mean square error, *MI* mutual information,

*PANSS* positive and negative syndrome scale, *BPRS* Brief Psychiatric Rating Scale, *DSM* Diagnostic and Statistical Manual of Mental Disorders, *ICD* international classification of diseases, *CGI* clinical global impression, *QCA* quantitative clinical assessment, *PSP* personal and social performance scale, *SANS* scale for assessment of negative symptoms,

*SFG* superior frontal gyrus, *PreCG* precentral gyrus, *InsOperc* insular–opercular cortex, *PCG* postcentral gyrus, *STC* superior temporal cortex.

### Clinical characteristics

The sociodemographic and clinical characteristics of participants in included studies are presented in Table 1. Among the 28 included publications, the majority of them considered only subjects with the first episode of psychosis (FEP) [23, 28–37], while 10 studies considered patients with chronic SCZ [38–47]. The remaining selected studies included both FEP and SCZ patients [48–54].

Regarding the pharmacological treatment that was used to explore outcome prediction in patients with psychosis, in the broad majority of included studies patients were treated with SGAs. Only in 2 studies subjects were treated only with FGAs, while in four studies both classes of antipsychotics were considered.

### ML approaches

Importantly, the included studies used heterogeneous predictors and different ML approaches to develop models to predict treatment outcomes in individuals with psychotic disorders. Indeed, several sociodemographic, clinical, and neuroimaging measures have been used as variables, or features, for the ML analyses performed in the included studies.

Specifically, 23 studies used only one modality as input to the ML algorithms, for example only MRI or electroencephalography (EEG) data, while the other five studies combined or compared different modalities. In order to better analyze and compare different selected articles, in the present review results of single-modality studies and multi-modality studies have been presented separately in the following sections.

Among the single-modality studies, many of them used functional and structural brain features for developing an ML prediction model. Specifically, 6 studies used resting-state fMRI (rs-fMRI) data [29, 30, 33, 34, 52, 53] while 1 study analyzed task-based fMRI images [39]. Moreover, three studies used sMRI measures [31, 49, 50], three studies used EEG data [41, 43, 47], one study considered positron emission tomography (PET) data [54], and one study used proton magnetic resonance spectroscopy (MRS) data [36]. Conversely, eight studies used clinical and sociodemographic data as input features to the ML algorithms [23, 35, 37, 38, 40, 42, 44, 51]. Among the multi-modality studies, different sets of features were used together as input to the ML algorithms. Specifically, Cui et al. [48] used both sMRI and fMRI data, Wang et al. [46] used a combination of sMRI, fMRI, and cognitive data, Ambrosen et al. [28] considered sMRI, EEG, and cognitive data together, while Uematsu & Hisanobu [45] used sMRI and sociodemographic data. Finally, Ebdrup et al. [32] used sMRI, EEG, DTI, and neurocognitive test data separately for ML analyses.

Additionally, included studies differed in clinical outcomes that were used in the ML analyses. The broad majority of them used changes in scores of psychotic symptom severity scales, such as the Positive and Negative Syndrome Scale (PANSS) and the Brief Psychiatric Rating Scale (BPRS), as measures of clinical improvement [28–31, 33–35, 39, 41, 43, 44, 46, 48–50, 53, 54]. In many of these studies, ML methods were applied to predict binary outcomes (“response” versus “no response”, or “remission” versus “no remission”) to antipsychotic treatment [28, 29, 32, 35, 39, 41, 43, 44, 46, 48, 49, 51, 54] while several studies used ML techniques for predicting continuous values such as reduction rates of PANSS and BPRS. Additionally, as the definition of treatment outcome is not merely defined by the symptomatic response, but might base on complex clinical and social domains, other measures and scales assessing specific symptoms and functioning criteria were used as labels in many studies [23, 36–38, 40, 42, 45, 47, 51, 52]. Therefore, the heterogeneity and complexity of relevant outcome indicators pose a challenge in synthesizing results and generating robust overall conclusions of the relevance of the included studies. Moreover, the use of different outcome measures influenced the type of ML methodology applied to develop accurate and generalizable treatment prediction models.

Indeed, several ML methods have been applied to develop accurate and generalizable treatment prediction models. Different ML approaches used in studies included in this review could be divided into classification algorithms, generally used to categorize the data into different outcome classes, and regression algorithms, performed to predict a continuous outcome value based on the input features. Specifically, classification algorithms were operated by 14 studies [23, 32, 35, 37, 41, 43, 44, 46–49, 51, 52, 54] while regression algorithms were used by 12 studies [29–31, 33, 34, 36, 38, 40, 42, 45, 50, 53]. Additionally, two studies used both classification and regression algorithms to predict symptomatic improvement in patients with psychosis [28, 39].

Finally, a difference between studies regarding methodology was in the use of single or multiple ML approaches. Specifically, 17 studies used only one ML method to identify specific predictors and develop an accurate treatment prediction model [29–31, 33–37, 42, 45–50, 52, 53], while the remaining studies compared the accuracy of different ML methods [23, 28, 32, 38–44, 51, 54]. Specifically, several studies found that support vector regressor (SVR) has the highest and most consistent overall accuracy compared to different ML techniques [23, 41, 43, 54], suggesting that SVR can deal with imbalanced datasets more effectively than other ML approaches [23, 41]. Nevertheless, other studies showed that predictions on the same dataset were similar regardless of the different ML methods used [28, 32, 40, 51], while in one study a random forest algorithm performed best relative to other approaches [44]. Finally, one study that compared different ML methods and one deep learning (DL) approach showed that the best overall performances were achieved using DL, suggesting that DL might be a promising approach for treatment predictions in patients with psychosis and supporting the development of DL-based methods in future research [39].

### Summary of single-modality studies

As previously reported, the broad majority of included studies used features extracted from a single modality as input to the ML algorithms. Many of them used neuroimaging measures to predict treatment outcomes in patients with psychosis, while others used clinical features.

#### sMRI features

Only three studies used structural neuroimaging features as input to the ML algorithms. Cui et al. [49] analyzed a cohort of 191 FEP and SCZ subjects, who were classified as responders or non-responders to antipsychotic treatment based on the reduction of PANSS scores. The input to the random forest (RF) classifier were different thalamic morphological features, such as volume and thickness. ML analyses showed that antipsychotic treatment response was predicted with an accuracy of 75%, suggesting that thalamus radiomic features can be promising in the definition of treatment selection.

Another sMRI study analyzed data of 44 FEP individuals treated with olanzapine for 8 weeks [31]. The gray matter volumes (GMV) of subregions of the superior temporal gyrus (STG) were used as input to an SVR, and the reduction rate of PANSS total score was the outcome measure. The SVR results exhibited a significant association between GMV of the STG and symptomatic improvement as effect of antipsychotic treatment.

Finally, Homan et al. [50] conducted an sMRI study on 82 FEP and early-diagnosed SCZ subjects treated with aripiprazole or risperidone for 12 weeks. They performed a partial least squares (PLS) regression using nodal degrees, calculated from brain cortical thickness, as predictors, and the continuous treatment response, based on the BPRS reduction, as output. Results of PLS regressions suggested that nodal degree in orbito- and prefrontal areas significantly contributed to the prediction of treatment response, with additional contributions from superior temporal regions.

In summary, these studies demonstrate structural radiomics approaches to predict the clinical response of antipsychotic treatment in early SCZ with significant accuracies. Specifically, GMV and thickness measures of specific brain regions, such as thalamic, temporal, and frontal areas, may represent important features that could play a role in the development of prognostic tools for individualized early treatment of SCZ [31, 49, 50].

#### fMRI features

Among the seven included fMRI studies, most of them considered resting-state activity and functional connectivity (FC) measures as predictive features, while only one used task-based functional brain activations as predictors. These studies showed substantial heterogeneity in the brain functional biomarkers that were found as meaningful predictors of antipsychotic treatment outcomes. Specifically, Blessing et al. [29] recruited 29 FEP subjects treated with SGAs for 8 weeks. They explored whether baseline brain functional connectivity (FC) predicted treatment response using the RF algorithm. ML analyses showed that hippocampal FC with insular–opercular cortex, superior frontal gyrus, precentral gyrus, and postcentral gyrus predicted treatment response with an accuracy of 89%.

Li et al. [33] recruited two independent samples of FEP patients treated with olanzapine for 8 weeks: one sample of 32 subjects as a training set and another sample with 44 subjects as a test set. The fractional amplitude of low-frequency fluctuation (fALFF) and SVR analysis were used to predict treatment response, showing a positive relationship between baseline fALFF levels in the left ventromedial putamen and improvement in positive symptoms. Moreover, the same SVR analyses were performed using global-brain FC (GFC), revealing a positive relationship between GFC in the bilateral anterior cingulate cortex and improvement in negative symptoms [34].

Also, Sarpal et al. [52] divided the cohort into a training set of 41 FEP subjects and a test set of 40 patients with chronic SCZ to develop a prognostic index based on rs-fMRI. A Cox regression analysis was performed in subjects classified as responders and non-responders. A striatal connectivity index was built from FC values between the striatum and 91 brain regions. The index significantly predicted the treatment response, and this result was validated in the independent cohort of antipsychotic-treated patients.

Shan et al. [53] explored whether the brain voxel-mirrored homotopic connectivity might predict individual treatment response in 21 patients with SCZ treated with olanzapine. An SVR analysis revealed that FC in the superior/middle prefrontal cortex at baseline could predict the symptomatic improvement of PANSS total, positive, and negative symptom subscale scores after 8 weeks of treatment.

Cao et al. [30] enrolled a small dataset of 43 FEP subjects for 10 weeks of risperidone treatment. By using SVR analysis and the FC of the superior temporal cortex with dorsal-lateral prefrontal, cingulate, temporal, and parietal cortices, this study predicted response to antipsychotic treatment at an individual level with an accuracy of 82.5%.

Finally, Smucny et al. [39] evaluated the ability of different ML and DL algorithms to predict symptomatic improvement in 65 patients with SCZ and 17 patients with bipolar disorder treated with SGAs by using fMRI frontoparietal activations during a continuous performance task as features. Higher overall performances were obtained using DL (accuracy of 70%) compared to ML algorithms, suggesting that DL might be a promising approach to predict treatment outcomes in SCZ.

In summary, despite a wide degree of methodological heterogeneity between the included studies, these findings suggest that fMRI features may contribute to the prediction of clinical outcomes in the early onset of psychosis with high accuracies. Specifically, several FC studies revealed that brain areas implicated in functional networks that play a key role in emotion and cognitive regulation were the most predictive features of treatment outcome and may be targets of antipsychotic treatment [29, 30, 34, 52, 53]. Moreover, it was found that also specific patterns of brain activation in cortical and subcortical brain regions may be useful for predicting treatment outcomes in the recent onset of psychosis [33, 39]. However, it is important to note that included studies presented several limitations, such as small sample sizes [29, 30, 33, 34, 39, 52, 53] and lack of replication samples [29, 30, 34, 39, 53].

#### Other neuroimaging features

Among studies that used other neuroimaging techniques as features for their ML analysis, Veronese et al. [54] used a PET approach to identify potential brain functional biomarkers of treatment stratification in 26 FEP subjects and 24 non-responsive SCZ patients. Linear and nonlinear ML analyses were performed; higher predictive power of treatment response was shown by linear SVM with an area under the curve (AUC) of 0.89 for striatal biomarkers, a good result that needs to take into consideration the small, but balanced, dataset.

Wood et al. [36] used proton MRS to investigate the predictive value of frontal and temporal spectroscopy measures in a modest dataset of 46 FEP patients treated with SGAs. An omnibus multivariate regressor was used to predict clinical and functional outcomes. It was found that *N*-acetylaspartate and choline-containing compounds to creatine and phosphocreatine ratio in the prefrontal cortex were significant predictors of antipsychotic treatment response.

#### EEG features

Interestingly, the three studies that used EEG features aimed to perform ML classification analysis on chronic treatment-resistant SCZ subjects treated with clozapine. Firstly, Khodayari-Rostamabad et al. [47] enrolled 37 subjects to conduct a kernelized PLS regression procedure, showing that a set of pre-treatment discriminative EEG features was able to predict clinical response to clozapine with an accuracy of 85%. The other studies that aimed to develop a prognostic algorithm based on pre-treatment EEG data compared different ML approaches. They found that SVM resulted to be the method with the highest accuracy (95.83% [41] and 89.90% [43] respectively) in discriminating between responders and non-responders to clozapine treatment. Should be noted that all three studies considered a small dataset, which was also unbalanced in one study [41].

#### Socio-demographical and clinical features

Noteworthy, several studies used different sociodemographic and clinical measures as features for ML analysis. Many of them found that important predictors of antipsychotic treatment outcome were baseline severity of psychotic symptoms [35, 37, 42, 44, 51] and comorbidities [23, 40], suggesting that ML models developed by including routinely available, patient-reportable information might present adequate predictive ability to be applied in clinical settings.

Most of these studies aimed to identify baseline measurable socio-demographical and clinical characteristics to predict treatment outcomes in chronic SCZ. Specifically, Anderson et al. [40] recruited 763 chronic SCZ patients and assessed baseline clinical predictors of treatment response using multivariate Cox and generalized linear regressions. The AUC of an ensemble of different models resulted in 0.75 for clinical remission. The most important predictors of remission were the use of long-acting injectable antipsychotics and the absence of psychiatric comorbidities.

In a study assessing 242 chronic SCZ patients treated with clozapine, the Least Absolute Shrinkage, and Selection Operator (LASSO) regression was used to develop a response prediction model [42]. The predictors of response to clozapine were severity of illness at baseline, female gender, and comorbid mood disorder.

Moreover, Legge et al. [38] recruited 1070 subjects with chronic SCZ or related psychotic disorders to investigate the ability of demographic and clinical premorbid factors to predict resistance to antipsychotic treatment. The authors performed a multivariate logistic regression and a conditional inference forest model, finding that earlier age at the onset of psychosis and poor premorbid functioning might be useful predictors of antipsychotic treatment outcome.

Additionally, many studies have found that measures of psychotic symptom severity at baseline might be considered adequate clinical predictors of antipsychotic treatment outcomes in patients with SCZ. Indeed, PANSS scores at baseline resulted to be the most predictive features in a study that enrolled 639 SCZ [44]. The RF algorithm performed best relative to other ML classifiers (SVM, LR, and Naïve Bayes) in terms of model ability to classify patients’ responses after 6 months of treatment, showing an AUC of 0.7 on the test set.

Moreover, Li et al., [51] compared different ML approaches (logistic regression, stochastic gradient descendent, gradient boosting decision tree, extreme gradient boosting, and RF) to predict social functioning improvement in 550 patients with SCZ treated with SGAs. The best AUC (0.86) was reached with RF, while the most important features were the use of mood stabilizers, social functioning, and PANSS total scores at baseline.

Furthermore, some studies used socio-demographical and clinical features to predict treatment outcome in FEP patients. Specifically, in a multicenter study that recruited two independent samples of 334 FEP patients in the training set and 108 FEP patients in the test set, pre-treatment clinical information, particularly psychosocial, sociodemographic, and psychometric variables were used as features for ML analysis to predict functional outcome after treatment with FGAs or SGAs [23]. Several algorithms, such as linear and nonlinear SVM, decision trees, and univariate and multivariate logistic regression were compared, obtaining the highest balanced accuracy (71.7%) by using nonlinear SVM on the test set. The most valuable predictors identified were largely psychosocial features, rather than symptom data: unemployment, poor education, functional deficits, and unmet psychosocial needs.

Soldatos et al. [35] recruited 270 FEP subjects and used SVM algorithms to classify remission and non-remission in patients treated with SGAs. Using items from validated clinical scales assessing psychotic symptom severity and functioning as features, the ML model significantly predicted early remission after treatment.

Finally, Wu et al. [37] used an ML method to develop antipsychotic treatment recommendations based on socio-demographical and clinical characteristics from 32277 FEP subjects. By using the individualized treatment rule (ITR) developed by applying a minimum loss–based ensemble ML method, the estimated treatment success rate was 51.7%. Results of this study suggested that an ITR developed using demographic and clinical predictors such as age, psychotic symptom severity, and the use of mood stabilizers or benzodiazepines, may be associated with an increase in the treatment success rate among FEP subjects.

### Summary of multi-modality studies

In recent years, advances have been made toward combining data from multiple modalities, in order to improve treatment response prediction. In this review, we included five studies applying multimodal approaches to patients with psychosis for treatment response prediction. These studies have utilized features from a variety of modalities, including structural and functional neuroimaging, socio-demographical, and cognitive data.

Specifically, Ambrosen et al. [28] analyzed a cohort of 138 initially drug-naïve FEP patients. They used sMRI, EEG, and cognitive data in two independent ML approaches, one based on a single algorithm and the other incorporating an ensemble of algorithms. For the prediction of short- and long-term antipsychotic treatment response, ML analyses yielded non-significant results. Moreover, in another study assessing treatment response in 46 antipsychotic-naïve FEP patients by using EEG, sMRI, and neurocognitive features, none of the SVM algorithms predicted symptom remission [32].

Cui et al. [48] used sMRI and fMRI features to predict the early response to antipsychotic treatment in 148 SCZ subjects. The SVM method was used to construct the classification model based on LASSO features. The prediction accuracy was 80.38% for the model using fMRI features only, 69.68% for the model using sMRI features, and 85.03% for the model combining both features, highlighting the importance of multimodal neuroimaging approaches in treatment response prediction.

Uematsu & Hisanobu [45] employed multiple regression analysis to predict treatment response based on sMRI and socio-demographical features in 40 SCZ patients. The results of this study demonstrated that the size of the cerebellar vermis and specific clinical features, such as symptom severity rates and duration of hospitalization, were related to antipsychotic treatment response.

Finally, Wang et al. [46] recruited 57 SCZ patients to assess whether neuroimaging and genetic features are predictive of antipsychotic treatment outcomes. The extreme gradient boosting (XGBoost) ML method was employed to combine sMRI, fMRI, and the schizophrenia polygenic risk score (PRS) as predictive features. Considering the small dataset, the combination of MRI measures (especially fALFF, GMV, and surface curvature) and PRS predicted treatment response with an accuracy of 86%, suggesting the importance of neuroimaging and genetic predictors in ML studies.

In conclusion, studies that compared different features found that functional neuroimaging contributed the most to predictions of clinical outcomes of psychosis relative to specific structural neuroimaging [46, 48] and genetic features [46]. Nevertheless, most of the models that combined multiple features showed higher accuracy than single-modality models, suggesting that, due to the complexity and heterogeneity of psychotic disorders, multimodal approaches may be able to predict more accurate outcomes of antipsychotic treatment [46, 48]. However, these findings must be interpreted with caution due to inconclusive results reported in some of the included studies [28, 32].

## Discussion

Prediction of treatment outcomes remains a significant challenge for psychiatry in the current era of personalized medicine. The increasing availability of large-scale datasets contributed to the development of sophisticated ML approaches, which have improved the accuracy of prediction over the use of conventional statistical models by capturing complex, nonlinear relationships in the data [55]. In recent years, ML methods have been seen as a promising approach for the automatic and robust prediction of treatment outcomes in psychosis. In this context, several studies have been conducted to identify predictive biomarkers that may contribute to direct the choice of antipsychotic treatment in patients with psychotic disorders.

The present article offers a narrative review of the original research studies that made use of ML approaches and multi-source features to predict clinical outcomes in patients with FEP or chronic SCZ treated with antipsychotics. The purpose of this review was to explore the role of various ML methods to determine suitable biomarkers for outcomes in the treatment of psychosis. Advancements in neuroimaging, electrophysiology, genetics, and clinical and cognitive testing in recent years have opened opportunities for the identification of quantitative biomarkers that may help the prediction of clinical outcomes in psychosis.

The majority of included studies considered structural and functional neuroimaging biomarkers as predictive features used in ML analysis. Indeed, in recent years ML approaches have been successfully applied for the analysis of neuroimaging data for the investigation of SCZ [56]. It is important to note that, despite considerable clinical and analytical heterogeneity, most of the reviewed studies found that the use of neuroimaging biomarkers predictors was associated with the high accuracy of ML models. Specifically, several studies showed that resting-state FC measures could represent effective predictive biomarkers of antipsychotic treatment efficacy. This is in line with the results of a recent meta-analysis showing that rs-fMRI network segregation and integration metrics are consistent determinants of treatment response in SCZ [57].

Additionally, we included studies investigating other neurobiological markers, such as electrophysiological and genetic data, as predictors of treatment response in psychosis. Interestingly, in line with the fMRI evidence, studies that used pre-treatment EEG data as features found significant accuracy in the prediction of clinical response to clozapine therapy in treatment-resistant SCZ, suggesting that EEG features might be effective predictors of response to antipsychotic treatment in patients with specific characteristics [43]. Moreover, although genome-wide genotype data show promise in aiding clinical decision-making in psychiatry [58], only one study has found a potential contribution of polygenic risk scores in the prediction of antipsychotic medication treatment outcomes in SCZ [46].

It is important to mention that many studies investigated potential sociodemographic and baseline psychopathological predictors of antipsychotic treatment outcomes in psychosis. Included studies showed that clinical features, such as specific sociodemographic characteristics, symptom severity, and comorbidities, allow individualized prognostic predictions in people with SCZ or FEP. Noteworthy, establishing robust and generalizable clinical predictors of response to antipsychotics could improve the pathophysiological understanding of SCZ and the development of new treatments based on patient characteristics [59]. Moreover, accessible clinical predictors could have implications for identifying patients that would likely benefit from specific treatments, thus enhancing the personalized management of patients [60].

Nevertheless, although single-modality features allow individualized prognostic prediction with significant accuracy, this accuracy may not be sufficient for translation in real-world clinical practice. In recent years, several studies suggested to improve predictive value by combining information from different types of data using a multi-modality ML framework [61–63]. Specifically, most of included studies combined MRI measures with other neuroimaging, genetic, or clinical features. Examining the additive effects of combining two or more types of features, the predictive value of neuroimaging metrics could be improved [46, 48]. Although these findings must be interpreted with caution, evidence from most of the reviewed studies applying multimodal ML supports the utility of combining multiple features to provide comprehensive information for the personalized management of psychotic patients.

However, heterogeneity in ML methods, prognostic features, treatment approaches, and clinical presentation complicate the identification of robust and reproducible clinical and biological markers of treatment outcomes in psychosis [59]. In this context, several factors need to be taken into consideration as they influence the performance of the classifiers, limiting the joint interpretation of the study findings.

Specifically, approximately half of the studies reviewed employed less than 60 subjects, which is a limitation because a small sample size could lead to overfitting, showing a high accuracy that would not be replicated when the model is applied to a new dataset. As shown by the studies reviewed, the ones with the lowest accuracies were the ones with the biggest dataset. Nevertheless, good performance was reached even with big datasets showing that ML can be used to reliably predict the treatment outcome. Thereby, dataset size should be taken into consideration by researchers when applying ML algorithms to their data showing realistic performance measures. Also, when using classifiers, classes should be balanced, otherwise, the accuracy could be misleading. In our study, most works with unbalanced datasets overcome this problem using balanced accuracy as a performance measure to evaluate classifiers. Finally, an independent dataset should be used to test the classifiers and show the potentiality and replicability of the algorithm. Most of the studies reviewed did not employ a test set, while its importance is clear when the accuracy measures for both the training and the test set are reported showing a lower accuracy for the test set [35, 37, 49]. Among the limitations of the studies reviewed, there is the absence in some cases of quantitative performance measures like precision, sensitivity, and specificity. Besides, it should be considered that an important issue of the ML approach is to choose the suitable dataset to be analyzed to predict the outcome of interest and since there are not any studies reporting negative results, we can speculate that papers tend to be published only when they show some consistent results. Therefore, unreliable datasets are usually dismissed and not reported in the literature; this is a bias that needs to be considered.

Finally, prognostic factors of treatment outcome were investigated at different phases of psychosis. Indeed, many studies used ML methods to identify antipsychotic outcome predictors in drug-naïve FEP patients, providing computational models to optimize the treatment at the early stage of SCZ. Notably, increase treatment efficacy and reduce adverse effects of antipsychotics is a crucial task in individuals with FEP, with a relevant impact on illness outcomes [64, 65]. Conversely, several studies used ML techniques to predict treatment response in the chronic course of SCZ. Remarkably, considering that chronic SCZ has a high burden for patients and healthcare services, the identification of prognostic factors that could help clinical management and treatment effectiveness is important to prevent illness progression [66]. However, differences in clinical manifestations and a large number of features must be evaluated, and many treatment strategies have to be tested. In this context, recent research has highlighted the importance of outcome prediction in treatment-resistant SCZ [67]. Several studies included in this review focused on clinical and neurobiological predictors of treatment-resistant SCZ outcomes, showing that ML approaches may improve the prediction accuracy of responsiveness to clozapine treatment [41–43].

In conclusion, future research in this field should create prediction models presenting adequate predictive ability applied at different stages of SCZ illness. Indeed, SCZ and psychotic disorders are among the principal causes of global disability and are also associated with significant economic costs for patients, caregivers, and society overall [54, 68]. A recent study highlights that the total estimated burden of SCZ doubled in the last years [68] and its cost is estimated to be greater than the annual costs of all cancers combined [69]. Antipsychotic drugs are central to treatment, but clinicians are currently unable to predict response using standard clinical interviews [46], which contributes to relatively low response rates, longer and more frequent hospital stays, and an increase in overall disease burden. Hence, the development of more sophisticated predictive methods to be used in everyday clinical practice seems necessary to optimize treatment plans as early as possible. Although this is likely to increase clinical management costs in the short term, effective prediction tools would certainly reduce overall costs over a medium-to-long timeframe. Indeed, the inclusion of methods such as MRI or other neurobiological markers to rapidly optimize treatment plans would lead to a reduction of relapses over time, a lower hospitalization rate, and an improved course of illness for the patient, which ultimately reduces costs for the health system.

### Limitations

Several limitations need to be considered when interpreting the findings of this review. First, included studies were heterogeneous in design, choice of prognostic features, ML algorithms, implementation, and result validation. These methodological differences must be taken into consideration when evaluating and comparing predictive models. Second, many studies used relatively small samples, especially those that considered electrophysiological and neuroimaging features as input to the ML algorithms. Indeed, small datasets can lead to the overfitting of ML models and produce results that will not replicate in independent samples. Third, many studies presented a lack of replication samples, limiting the validation of predictive models on independent cohorts. Furthermore, the heterogeneity of prognostic features limited the synthesis of results and the generation of robust overall conclusions. Finally, participants recruited in the included studies presented heterogeneous clinical characteristics (e.g., illness stage), which can require specific predictive biomarkers and treatment management. These factors should be carefully examined before recommending the use of clinical or neurobiological predictors of antipsychotic treatment outcomes in subjects with psychosis. Future studies need to focus on refining feature characterization to improve prediction accuracy, validate prediction models, and evaluate their implementation in clinical practice.

## Conclusion

This literature review examined the findings from ML approaches used to predict antipsychotic treatment outcomes in patients with psychosis. Many neuroimaging, neurophysiological, genetic, sociodemographic, and clinical features were identified as putative predictors of clinical outcomes in patients with FEP and chronic SCZ treated with antipsychotics. Despite considerable clinical and analytical heterogeneity, most of the included studies considering single-modality or multi-modality features predicted responses to antipsychotics with good accuracy. Interestingly, by examining the additive effects of combining multi-source features, the predictive value of ML models could be improved. However, heterogeneity among studies in terms of considered features, ML methodology, and clinical characteristics like stages of the illness complicate the identification of both clinical and biological markers of response, remission, and recovery after antipsychotic treatment. In conclusion, although ML is a promising approach in the research of treatment outcome prediction in psychosis, further research is required to identify the actual benefits of ML in this area. With ML tools becoming more accessible for researchers and clinicians, it is expected that the field will continue to grow and that novel applications will follow.
